# Supplementary material for: Novel insights into breast cancer copy number genetic heterogeneity revealed by single-cell genome sequencing
Source: eLife. 2020 May 13;9:e51480. doi: 10.7554/eLife.51480 (PMC7220379; doi:10.7554/eLife.51480)
Supplement: Transparent reporting form [file elife-51480-transrepform.pdf]

eLIFE
1st Floor
24 Hills Road
Cambridge CB2 1JP, UK
P 01223 855340
W elifesciences.org
T @elife

eLife's transparent reporting form

We encourage authors to provide detailed information *within their submission* to facilitate the interpretation and replication of experiments. Authors can upload supporting documentation to indicate the use of appropriate reporting guidelines for health-related research (see EQUATOR Network), life science research (see the BioSharing Information Resource), or the ARRIVE guidelines for reporting work involving animal research. Where applicable, authors should refer to any relevant reporting standards documents in this form.

If you have any questions, please consult our Journal Policies and/or contact us: editorial@elifesciences.org.

Sample-size estimation

- You should state whether an appropriate sample size was computed when the study was being designed
- You should state the statistical method of sample size computation and any required assumptions
- If no explicit power analysis was used, you should describe how you decided what sample (replicate) size (number) to use

Please outline where this information can be found within the submission (e.g., sections or figure legends), or explain why this information doesn't apply to your submission:

Sample size (i.e. number of patient samples analyzed and # of single-cells sequenced per patient tumor) for the study was not estimated using any statistical methods as the design of the study at inception was exploratory – i.e. inferring novel insights from data which previously was not available. The only criteria used in selecting samples for intial processing and analysis was: equal representation of transcriptionally defined breast cancer subtypes (i.e. Luminal A, Luminal B, HER2, and Basal).

Replicates

- You should report how often each experiment was performed
- You should include a definition of biological versus technical replication
- The data obtained should be provided and sufficient information should be provided to indicate the number of independent biological and/or technical replicates
- If you encountered any outliers, you should describe how these were handled
- Criteria for exclusion/inclusion of data should be clearly stated
- High-throughput sequence data should be uploaded before submission, with a private link for reviewers provided (these are available from both GEO and ArrayExpress)

Please outline where this information can be found within the submission (e.g., sections or figure legends), or explain why this information doesn't apply to your submission:

Each single cell/nuclei sequenced technically represents a biological replicate from the analyzed biospecimen. Given the singularity of a "single-cell", there are no technical replicates (None of the single cell amplified DNA was sequenced twice).

In the process of employing highly parallel single-cell whole genome amplification and indexed sequencing of the resulting DNA (which was done in a format of 96 cells at a time – 96-well plate format processing), two outlier groups were noted.
Those are:
(1) cells for which, because of random sampling, sufficient sequencing depth was not achieved. The sufficient sequencing coverage has previously been described (Baslan et al, Genome Res 2015) - depth cut-off for dataset inclusion was 250,000 uniquely mapped reads. Cells not reaching this sequencing coverage were thus excluded from further analysis.
(2) cells for which an over-abundance of non-recurrent genomic segments with changes in copy number were observed. Those single-cells were removed from the datasets by employing the CORE algorithm (Krasnitz et al, PNAS 2013) to identify single-cells with recurrent genomic alterations (i.e. clonal cancer cells) for further study.
This is described in the Methods section of the manuscript.

All high-throughput single-cell sequencing data (as well as some bulk WGS data) has been deposited in a public repository (SRA), under accession PRJNA555560 and can be accessed.

Statistical reporting

- Statistical analysis methods should be described and justified
- Raw data should be presented in figures whenever informative to do so (typically when N per group is less than 10)
- For each experiment, you should identify the statistical tests used, exact values of N, definitions of center, methods of multiple test correction, and dispersion and precision measures (e.g., mean, median, SD, SEM, confidence intervals; and, for the major substantive results, a measure of effect size (e.g., Pearson's r, Cohen's d)
- Report exact p-values wherever possible alongside the summary statistics and 95% confidence intervals. These should be reported for all key questions and not only when the p-value is less than 0.05.

Please outline where this information can be found within the submission (e.g., sections or figure legends), or explain why this information doesn't apply to your submission:

All statistical analysis were performed using the R software package and described in the methods section in detail. A subset of the analyses are also noted in the corresponding figure legends where deemed appropriate.

(For large datasets, or papers with a very large number of statistical tests, you may upload a single table file with tests, Ns, etc., with reference to sections in the manuscript.)

eLife Sciences Publications, Ltd is a limited liability non-profit non-stock corporation incorporated in the State of Delaware, USA, with company number 5030732, and is registered in the UK with company number FC030576 and branch number BR015634 at the address 1st Floor, 24 Hills Road, Cambridge CB2 1JP | August 2014

Group allocation

- Indicate how samples were allocated into experimental groups (in the case of clinical studies, please specify allocation to treatment method); if randomization was used, please also state if restricted randomization was applied
- Indicate if masking was used during group allocation, data collection and/or data analysis

Please outline where this information can be found within the submission (e.g., sections or figure legends), or explain why this information doesn't apply to your submission:

Single-cells were grouped into two categories: normal or cancer cells. This was based on the absence or presence of recurrent copy number alterations, respectively.

Various patient groupings for association analysis with copy number heterogeneity are described in the methods section as well as the results sub-section "*CNA heterogeneity is associated with genetic, molecular, and clinical classifications".*

Additional data files ("source data")

- We encourage you to upload relevant additional data files, such as numerical data that are represented as a graph in a figure, or as a summary table
- Where provided, these should be in the most useful format, and they can be uploaded as "Source data" files linked to a main figure or table
- Include model definition files including the full list of parameters used
- Include code used for data analysis (e.g., R, MatLab)
- Avoid stating that data files are "available upon request"

Please indicate the figures or tables for which source data files have been provided:

All numerical data are derived and can be retrieved from the deposited publicly available sequencing data along with informatics pipelines/analysis detailed in the methods section. Summary statistics for variables such as approximate ploidy, % cancer composition, tumor size, and patient age, are listed in numerical format in Supplementary Figure 1D.
